# The effect of talus osteochondral defects of different area size on ankle joint stability: a finite element analysis

**DOI:** 10.1186/s12891-022-05450-2

**Published:** 2022-05-27

**Authors:** Jia Li, Yezhou Wang, Yu Wei, Dan Kong, Yuan Lin, Duanyang Wang, Shi Cheng, Pengbin Yin, Min Wei

**Affiliations:** 1 Department of Orthopedics, The First Medical Center, Chinese PLA General Hospital, Beijing, China; 2 National Clinical Research Center for Orthopaedics, Sports Medicine & Rehabilitation, Beijing, China; 3 Orthopedic Hospital, The First Affiliated Hospital of Nanchang University, Nanchang, China; 4 Department of Orthopedic Surgery, The Second Affiliated Hospital of Harbin Medical University, Harbin, China; 5 The Faculty of Orthopaedics, The Fourth Medical Centre, Chinese PLA General Hospital, Beijing, China

**Keywords:** Ankle injuries, Osteochondral lesion of the talus (OLTs), Finite Element Analysis (FEA), Ankle joint instability

## Abstract

**Background:**

Osteochondral lesion of the talus (OLT) is one of the most common ankle injuries, which will lead to biomechanical changes in the ankle joint and ultimately affect ankle function. Finite element analysis (FEA) is used to clarify the effect of talus osteochondral defects on the stability of the ankle joint at different depths. However, no research has been conducted on talus osteochondral defect areas that require prompt intervention. In this research, FEA was used to simulate the effect of the area size of talus osteochondral defect on the stress and stability of the ankle joint under a specific depth defect.

**Methods:**

Different area sizes (normal, 2 mm* 2 mm, 4 mm* 4 mm, 6 mm* 6 mm, 8 mm* 8 mm, 10 mm* 10 mm, and 12 mm* 12 mm) of the three-dimensional finite element model of osteochondral defects were established. The model was used to simulate and calculate joint stress and displacement of the articular surface of the distal tibia and the proximal talus when the ankle joint was in the heel-strike, midstance, and push-off phases.

**Results:**

When OLT occurred, the contact pressure of the articular surface, the equivalent stress of the proximal talus, the tibial cartilage, and the talus cartilage did not change significantly with an increase in the size of the osteochondral defect area when the heel-strike phase was below 6 mm * 6 mm. Gradual increases started at 6 mm * 6 mm in the midstance and push-off phases. Maximum changes were reached when the defect area size was 12 mm * 12 mm. The same patterns were observed in the talus displacement.

**Conclusions:**

The effect of the defect area of the ankle talus cartilage on the ankle biomechanics is evident in the midstance and push-off phases. When the size of the defect reaches 6 mm * 6 mm, the most apparent change in the stability of the ankle joint occurs, and the effect does not increase linearly with the increase in the size of the defect.

## Introduction

Ankle joint injuries account for 20% of joint injuries and represent a significant health care problem with a high recurrence rate [1]. More importantly, acute or recurrent ankle trauma is closely related to the occurrence of post-traumatic ankle osteoarthritis [2]. Osteochondral lesion of the talus (OLT) is one of the common ankle injuries [3, 4]. For OLT treatment, conservative management is primarily suitable for patients with mild clinical symptoms, small injury area, stable avulsion bone without fracture displacement, and chronic osteochondral injury in the talus, such as Hepple type I ~ II [5]. The surgical treatment should be considered when conservative treatment is noneffective for more than 3 to 6 months in the patients who have the Hepple type II ~ V symptomatic OLT [6–8]. The first-line treatment for OLT in clinical practice is bone marrow stimulation(BMS). The effect of BMS for more than 15 mm of defect is poor, and the effect for less than 10 mm of defect is more effective. Although BMS can promote cartilage repair, it can destroy the structure of the subchondral bone [9, 10]. BMS can cause additional damage when minor defects in the talus cartilage do not affect the stability of the ankle joint [11]. The size of defects diameter that do not require repair is clinically controversial [12]. There is no clear quantitative indicator of the size of the area to guide the selection of conservative or surgical treatment**.** Therefore, choosing the treatment method for OLT with different areas of defects is a complex problem for orthopedic surgeons [13].

For OLT, there are usually depth defects and area defects. Regarding the impact of depth defects, our research team found that the stability of the ankle joint is significantly affected when the depth of the defect exceeds 3 mm using finite element analysis (FEA) [14]. Currently, studies have revealed that the area of the OLT defect is an important factor affecting the ankle joint's stability and treatment effect [15]. Regarding the size of the area defect, the literature has shown that the defect with a diameter ≥ 15 mm is poorly treated with BMS [16]. However, no relevant research has clearly defined the impact of defects of less than 10 mm diameter on the stress and stability of the ankle joint. Therefore, understanding the impact of the size of the defect on the biomechanics and stability of the ankle joint is of great importance for the treatment of OLT defects in the talus.

By constructing a finite element model of the ankle joint, our group selected a normal cartilage model to simulate a depth of 1 mm size of the osteochondral defect of the talus. The area defect sizes are 2 mm * 2 mm, 4 mm * 4 mm, 6 mm * 6 mm, 8 mm * 8 mm, 10 mm * 10 mm, and 12 mm * 12 mm. FEA was performed to explore the influence of the size of defect areas on the biomechanics and stability of the ankle joint.

## Materials and methods

All patients provided informed consent and using of these data was conducted with the approval of our institutional ethical committee. All methods were carried out in accordance with relevant guidelines and regulations.

### Processing of the CT files and three-dimensional solid reconstruction

**Fig. 1:**
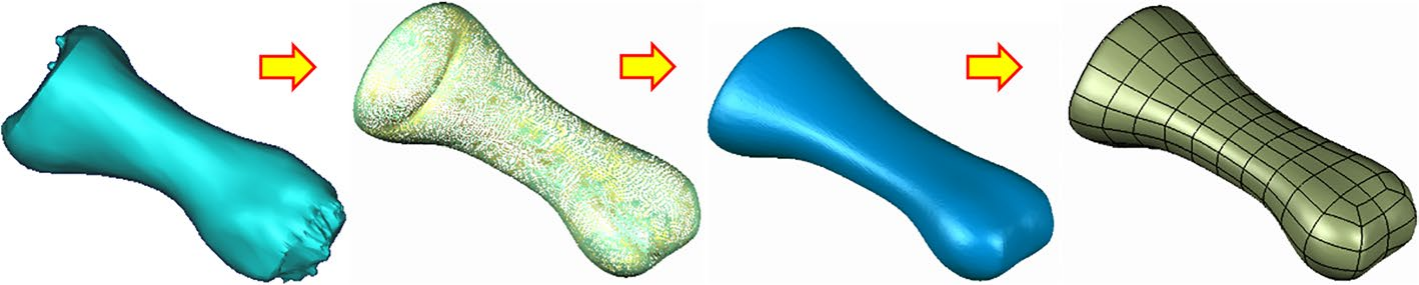
Diagram of image processing with Geomagic

An image of the right ankle joint of an adult man in a neutral position was obtained by computed tomography (CT) (64 slices, SIEMENS, USA). The thickness of the CT slice is 0.625 mm, and the bone threshold HU (Hounsfield Unit) is 0–1023. The image was input into the three-dimensional (3D) reconstruction software Mimics in the Dicom format to obtain a clear skeleton outline. After mask processing, the image was read in Geomagic (Geomagic, USA) using stereolithography (STL) format. Reverse engineering reconstruction was completed, and 3D graphics in the Initial Graphics Exchange Specification (IGES) file format was generated (Fig. 1).

### Construction of the working condition model

**Fig. 2:**
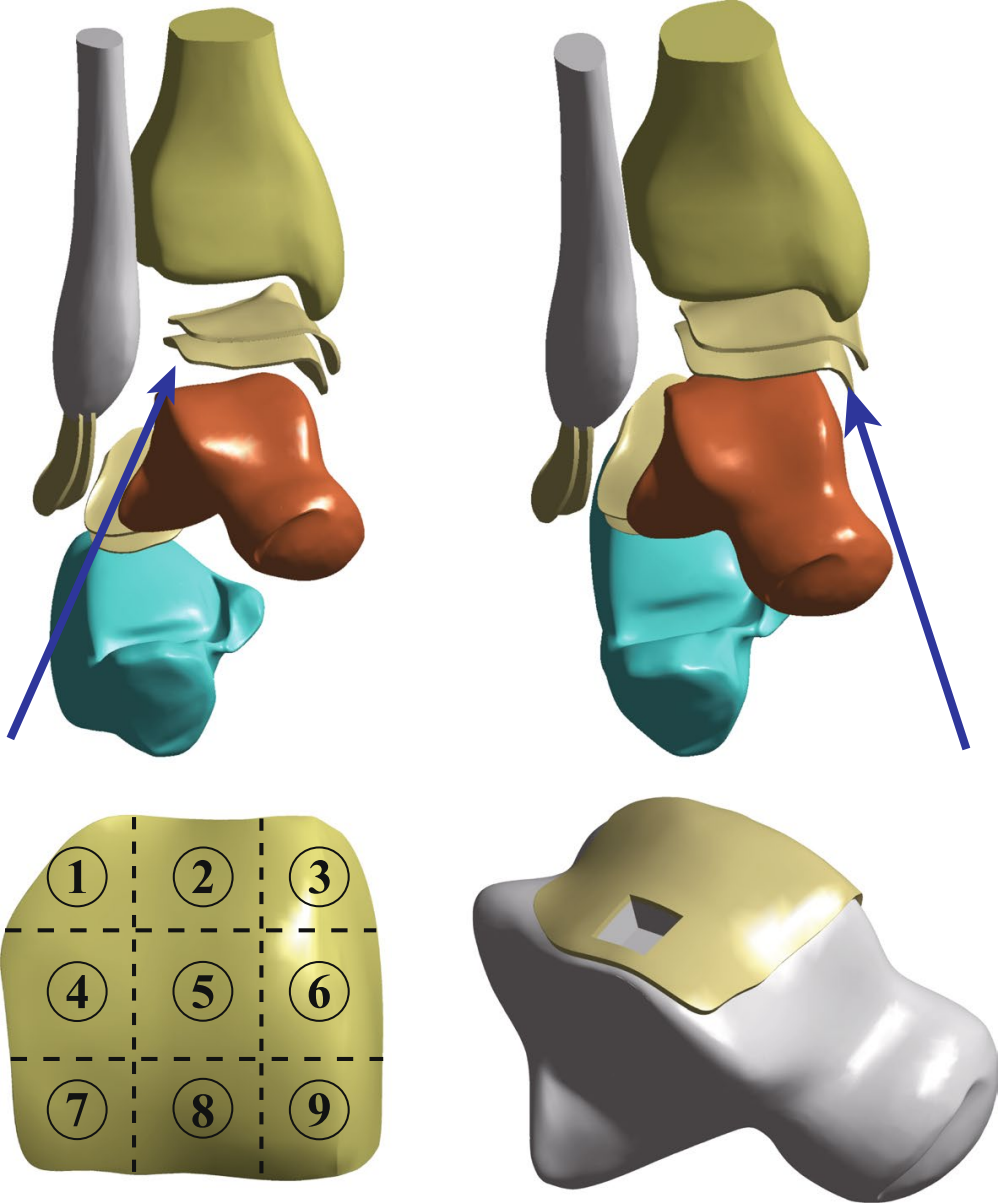
Location and size of OLT defect

After the foot skeleton and contour were built, all ligaments were connected with lines in the physiological position, and a complete foot model was generated. According to the anatomical data of the joint surface, cartilage boundaries were established, and cartilage joints were built with Geomagic with an offset thickness of 1 mm. Only the fibula and tibia of the calcaneal talus and the related cartilages and ligaments were required to be retained per the analysis requirements. Thus, a relatively complete 3D finite element model of the ankle joint of the normal adults was built. Based on the cartilage of the normal model, the talus cartilage was divided into nine regions using the nine-grid partition method. Studies have shown that area 4 is the most common area for talus cartilage injuries [17, 18]. This study simulated defects in cartilage and subchondral bones in region 4 of the talus. Because the existing literature did not study the area size of finite element and talus injury, we selected the following FEA: the experimental measurement depth 1 mm with the area defect sizes being 2 mm * 2 mm, 4 mm * 4 mm, 6 mm * 6 mm, 8 mm *8 mm, 10 mm *10 mm, and 12 mm * 12 mm (Fig. 2).

### Meshing

**Fig. 3:**
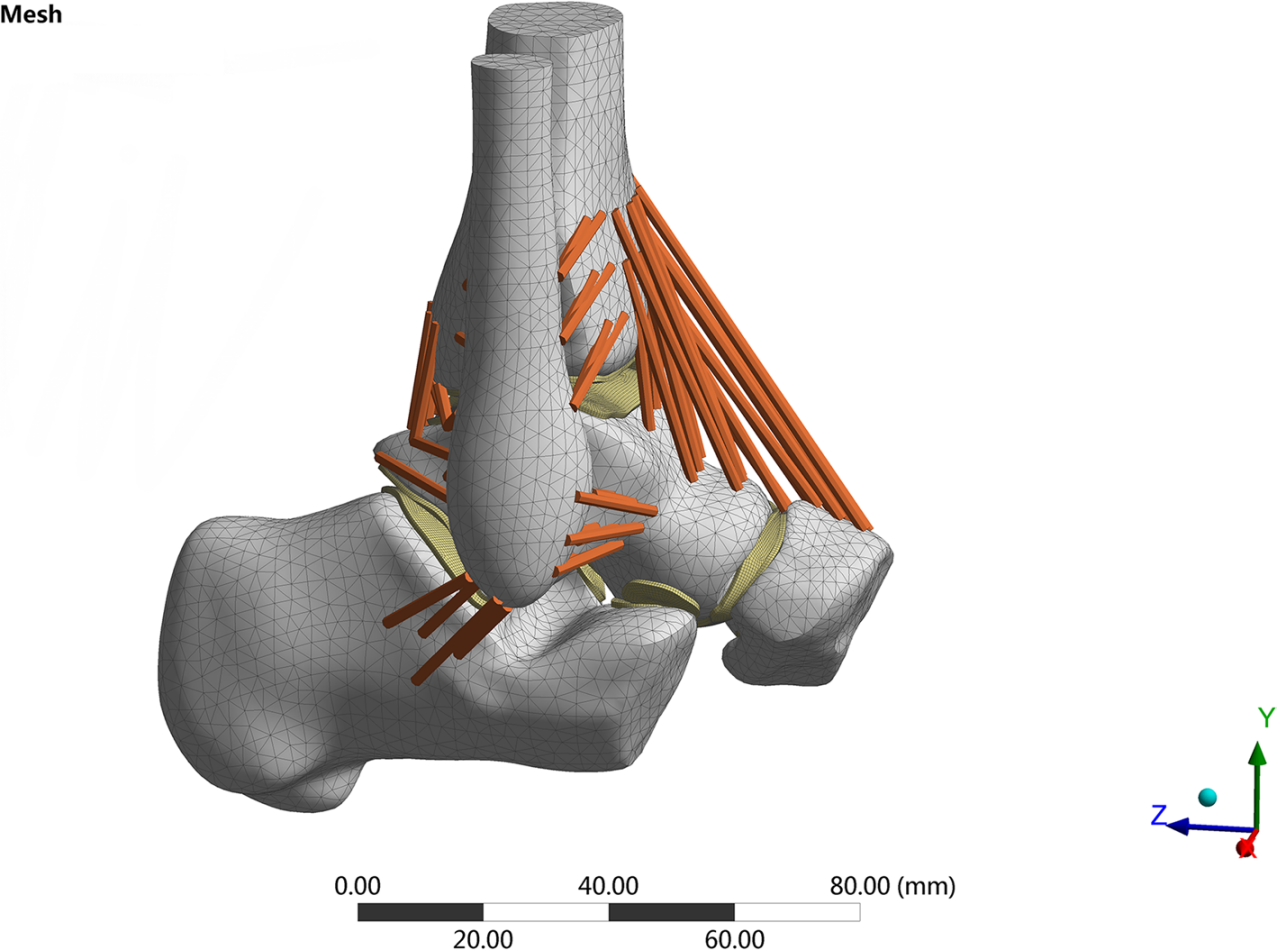
Mesh of ankle joint unit

The assembled solid model was imported into the Ansys Workbench (Ansys, USA). A Boolean operation was performed, material parameters were assigned, contact was defined, and the grid division process was completed. The solid unit comprised Solid 187 and Solid 95, and the ligament was a Link180 unit. Non-linear characteristics were set under tension without pressure (Fig. 3).

#### Material parameters and contact settings

All tissue materials involved in this model were simplified into isotropic homogeneous elastic materials. Material parameters are listed in Tables 1 and 2 [19–21].

**Table 1:** Properties of the bone and cartilage materials

Material	Modulus of Elasticity (MPa)	Poisson's ratio
Bone	7300	0.3
Cartilage	12	0.42

**Table 2:** Material properties of the ligaments

Ligament	Modulus of Elasticity (MPa)	Poisson's ratio	Sectional area (mm^2^)
AtiF	260	0.4	18.4
PtiF	260	0.4	18.4
AtaFi	255.5	0.4	12.9
PtaFi	216.5	0.4	21.9
CaTi	512	0.4	9.7
AtiTa	184.5	0.4	13.5
PtiTa	99.5	0.4	22.6
TiCa	512	0.4	9.7
TiNa	320.7	0.4	7.1

*AtiF* Anterior tibiofibular ligament, *PtiF* Posterior tibiofibular ligament, *AtaFi* Anterior talofibular ligament, *PtaFi* Posterior talofibular ligament, *CaTi* Calcaneofibular ligament, *AtiTa* Anterior tibial ligament, *PtiTa* Posterior tibial talus ligament, *TiCa* Tibiocalcanean ligament, *TiNa* Tibionavicular ligament

The contact settings between the components were set according to the actual condition. The cartilage was bound to the corresponding bones, and the friction coefficient between the articular surfaces of the cartilage was 0.01 [22, 23].

#### Applying loads and constraints

**Fig. 4:**
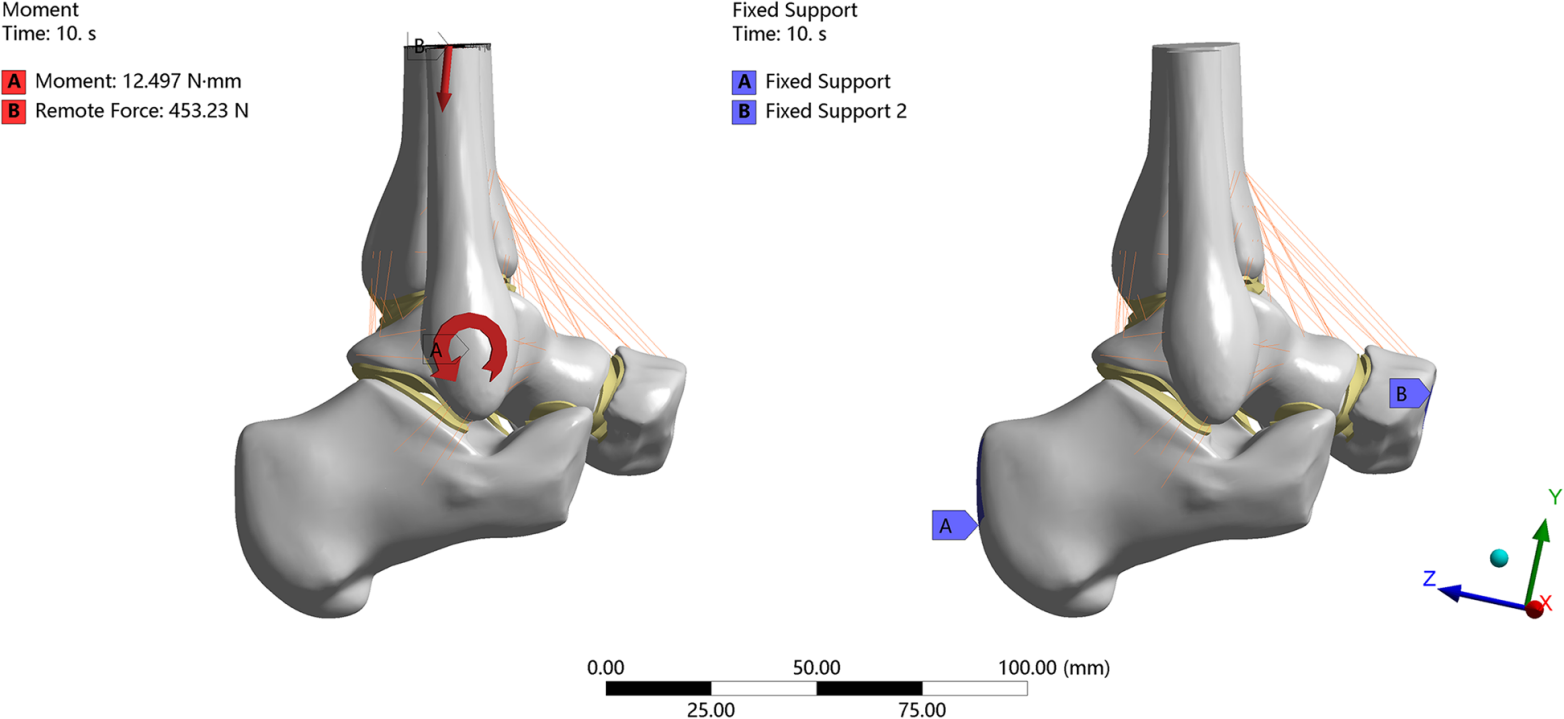
Diagram of constraint and load

The grid direction of the corresponding sites of the calcaneus and navicular bone was constrained so that the degree of freedom was 0. Three gait patterns were selected for analysis based on previous studies. As shown below, it was assumed that body weight was 600 N and foot length was 25.4 cm (Fig. 4). After the model was established, it was verified that the gait patterns were similar to those in previous studies [8, 14].

#### Experimental groups and data acquisition process

After the above model was developed, the following seven groups were established for the experiment: the normal osteochondral talus group and the groups with the area defect sizes of 2 mm * 2 mm, 4 mm * 4 mm, 6 mm * 6 mm, 8 mm *8 mm, 10 mm *10 mm, and 12 mm * 12 mm. In each group, the finite element method and the above model were used to simulate the stress on the ankle joint in the push-off phase, the midstance phase, and heel-strike phase to determine the contact pressure on the joint surface, the equivalent stress of the cartilage of the proximal talus and distal tibia in each phase and the displacement of the talus. The stress, contact state, and displacement of each component of the ankle joint were observed in the different groups to determine its maximum value and location. The maximum pressure was recorded as the experimental data and analyzed to obtain the column diagram, and the changes in pressure were discussed. In this study, the primary outcome was a displacement of the talus and contact pressure of the articular surface. Secondary outcomes were equivalent stresses of the proximal talus, tibial cartilage, and talus cartilage.

## Results

Using a 3D finite element simulation of osteochondral defects at different area sizes of the talus, the following was found:

### Contact pressure of the articular surface and displacement of the talus

The contact pressures of the normal ankle were 3.7599 Mpa, 4.8247 Mpa and 4.6199 Mpa in the heel-strike, midstance phase and push-off phases, respectively. There were no significant changes when the defect size was below 6 mm * 6 mm, including 2 mm * 2 mm (3.7737 Mpa 4.8719 Mpa, and 4.4613 Mpa, respectively) and 4 mm * 4 mm (3.9324 Mpa 5.0558 Mpa, and 4.5080 Mpa, respectively). The contact pressure began to increase gradually since the defect size at 6 mm * 6 mm (5.5525 Mpa and 5.3059 Mpa, respectively) in midstance phase and push-off phases. Stress reached the highest level in midstance phase and push-off phases when the defect size was 12 mm * 12 mm (8.7896 Mpa and 6.2716 MPa, respectively).

The displacements of the normal talus were 1.9665 mm, 5.8657 mm, and 5.3314 mm in the heel-strike phase, midstance phase, and push-off phase. There were no significant changes when the defect size was below 6 mm * 6 mm, including 2 mm * 2 mm (1.9814 mm, 5.9118 mm, and 5.3883 mm, respectively) and 4 mm * 4 mm (1.9866 mm, 6.0754 mm, and 5.5299 mm, respectively). The displacement of the talus began to increase gradually since the defect size at 6 mm * 6 mm (2.0156 mm,6.2910 mm, and 5.8229 mm, respectively)in three phases. The highest displacement was in the 12 mm * 12 mm group in three phases (2.6559 mm, 8.3045 mm and 7.3983 mm, respectively) (Table 3).

**Table 3:** Pressure of the ankle joint surface and displacement of the talus in the heel-strike phase, midstance phase, and push-off phases

Parameters	Contact pressure (Mpa)			Displacement of the talus (mm)		
	Heel-strike phase	Midstance phase	Push-off phase	Heel-strike phase	Midstance phase	Push-off phase
Normal	3.7599	4.8247	4.6199	1.9665	5.8657	5.3314
2 × 2 × 1	3.7737	4.8719	4.4613	1.9814	5.9118	5.3883
4 × 4 × 1	3.9324	5.0558	4.5080	1.9866	6.0754	5.5299
6 × 6 × 1	3.6558	5.5525	5.3059	2.0156	6.2910	5.8229
8 × 8 × 1	3.9323	6.3312	6.2716	2.2125	7.0086	6.3655
10 × 10 × 1	4.902	7.1888	7.3397	2.2968	7.6493	6.8185
12 × 12 × 1	4.6543	8.7896	9.6693	2.6559	8.3045	7.3983

### The equivalent stress of the proximal talus, tibial cartilage, and talus cartilage

In heel-strike phase, the normal equivalent stresses of the proximal talus, tibial cartilage, and talus cartilage were 2.106 Mpa, 1.6477 Mpa, and 2.2804 Mpa. Stress increased with increasing osteochondral defect area size. However, the stress did not change significantly, and the stress reached the highest level in the heel-strike phase when the defect size was 12 mm * 12 mm (2.2621 Mpa, and 2.6804 MPa, 3.0477 Mpa, respectively).

In midstance phase, the normal equivalent stress of the proximal talus, tibial cartilage, and talus cartilage were 4.4531 Mpa, 2.479 Mpa, and 2.7872 Mpa. Stress increased with increasing osteochondral defect area size. Stress reached the highest level in midstance when the defect size was 12 mm * 12 mm (8.3939 Mpa, and 8.9997 MPa, 8.5985 Mpa, respectively).

In push-off phase, the normal equivalent stress of the proximal talus, tibial cartilage, and talus cartilage were 3.1456 Mpa, 2.2873 Mpa, and 2.4853 Mpa. Stress increased with increasing osteochondral defect area size. Stress reached the highest level in midstance when the defect size was 12 mm * 12 mm (6.1956 Mpa, 8.1271 MPa, and 8.2563 Mpa, respectively) (Table 4).

**Table 4:** Equivalent stress of the proximal talus, tibial cartilage, and talus cartilage in the heel-strike phase, midstance phase, and push-off phases

Parameters	Equivalent stress of the proximal talus (Mpa)			Equivalent stress of tibial cartilage (Mpa)			Equivalent stress of talus cartilage (Mpa)		
	Heel-strike phase	Midstance phase	Push-off phase	Heel-strike phase	Midstance phase	Push-off phase	Heel-strike phase	Midstance phase	Push-off phase
Normal	2.106	4.4531	3.1456	1.6477	2.479	2.2873	2.2804	2.7872	2.4853
2 × 2 × 1	2.1009	4.4679	3.1722	2.311	5.0803	2.3641	1.8974	3.0599	2.9754
4 × 4 × 1	2.1125	4.617	3.246	2.3166	5.1477	2.4983	1.8261	3.5431	3.2516
6 × 6 × 1	2.129	5.1434	3.5119	1.6715	5.2665	2.9691	1.85	4.3454	4.1944
8 × 8 × 1	2.183	6.1385	4.3914	2.2682	5.5435	3.7991	2.4564	5.7413	5.2679
10 × 10 × 1	2.2712	7.5035	5.4651	3.1235	5.7034	4.0634	3.08	8.0266	6.8207
12 × 12 × 1	2.2621	8.3939	6.1956	2.6804	8.9997	8.1271	3.0477	8.5985	8.2563

## Discussion

The most important findings of the study were 1) in the heel-strike phase, the stress of the talus cartilage defect in different areas did not change significantly, and 2) in the midstance and push-off phases, when the defect area was below 6 mm * 6 mm, the changes in stress and displacement in each group were not obvious. The stress and displacement of the ankle joint had a significant positive trend of increase starting at the defect size of 6 mm*6 mm. When the defect area was 12 mm*12 mm, the changes reached maximum values in each group.

The rationality of the model was verified by comparing the peak pressure of the tibiotalar joint contact and the contact area in the intact model with previous biomechanical experiments and FE models. Parameters under three walking gaits of 600 N bodyweight were consistent with previous studies [21, 24–26] with the same loading condition, as shown in Table 5.

**Table 5:** Model validation to view the contact pressure and contact area between the tibiotalar articular surfaces

		Peak pressure of tibiotalar joint contact(MPa)	Contact area(mm^2^)
Heel-strike phase	Suckel, A ^24^	-	270
	Genfen ^21^	2.55	-
	Changhuai Lu ^25^	3.0	274.9
	**Our model**	**3.7599**	**263.6875**
Midstance phase	Suckel, A	-	415
	Hurschler ^26^	4.4	-
	Genfen	2.72	-
	Changhuai Lu	4.3	355.4
	**Our model**	**4.8247**	**358.8125**
Push-off phase	Suckel, A	4.8	335
	Genfen	3.55	-
	Changhuai Lu	4.8	250.7
	**Our model**	**4.6199**	**354.3125**

The surface of the ankle joint of the talus plays a vital role in the biomechanics of the ankle joint [27]. Previous studies have used biomechanic tests in cadaver bone to simulate defects of 6 mm, 8 mm, 10 mm, and 12 mm to clarify the effect of defects on ankle joint stress. The study only simulated a neutral position and a 15° plantar flexion position and found that when the defect was larger than 10 mm, stress gradually concentrated on the edge of the defect. This stress concentration could cause surgery failure when the area size was larger than 10 mm [28]. However, the study did not simulate the heel-strike phase, the midstance, and the push-off phase of the ankle joints and did not record the ankle stress and displacement changes when the OLT defect was at these positions. Clinically, for the treatment of OLT defects, the defect diameter less than 10 mm is an indicator [29–31]. Clinical symptoms require surgical intervention. However, there is no relevant research on the stress and displacement of the ankle joint with an area defect area smaller than 10 mm.

**Fig. 5:**
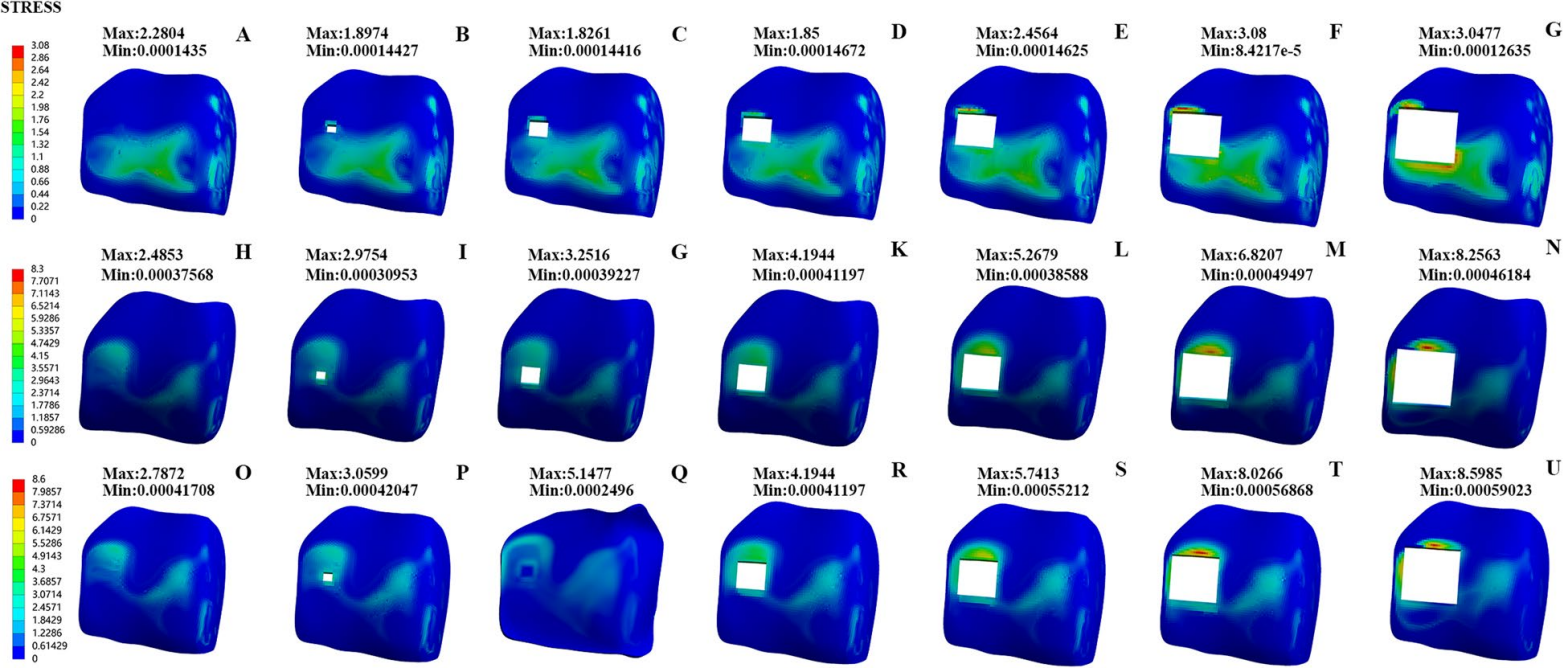
Comparison of the equivalent stress of talus cartilage of seven groups in the heel-strike phase (**A**-**G**), the push-off phase (**H**-**N**), and the midstance phase (**O**-**U**): Normal Group (**A**-**H**–**O**); 2 mm*2 mm defect (**B**-**I**-**P**); 4 mm*4 mm defect (**C**-**J**-**Q**); 6 mm*6 mm defect (**D**-**K**-**R**); 8 mm*8 mm defect (**E**-**L**-**S**); 10 mm*10 mm defect (**F**-**M**-**T**); 12 mm*12 mm defect (**G**-**N**-**U**)

Therefore, our group used the three-dimensional finite element mechanics of different OLT defects to simulate the changes in the stress and displacement of the ankle joint in the heel-strike phase, the midstance, and the push-off phase. We found that compared to normal talus cartilage when the area defect was less than 6 mm*6 mm, the equivalent stresses of the proximal talus, tibial cartilage, and talus cartilage (Fig. 5) did not change significantly compared to those of the normal talus cartilage. However, when the area size was greater than 6 mm*6 mm, stress increased with increasing area sizes of the defect in the midstance and push-off phases. The maximum stress could be increased more than 2–3 times but had little impact on the heel-strike phase. According to the motion mechanics of the ankle joint and the gait cycle, the heel-strike phase is when the heel touches the ground, which is the beginning of the support phase [32]. At this time, the front articular surface of the talus (area 1/2/3, Fig. 3) was in contact with the tibia, and the defect area was in zone 4 (Fig. 3), which was located in the middle medial part of the talus, and the stress was small. Therefore, the stress change was not evident in the heel-strike phase. In contrast, the stress in talus zone 4 increased and reached the maximum value in the midstance and push-off phases. The talus position is different during the movement of the ankle joint. We believe that this is why biological stresses have different tendencies.

**Fig. 6:**
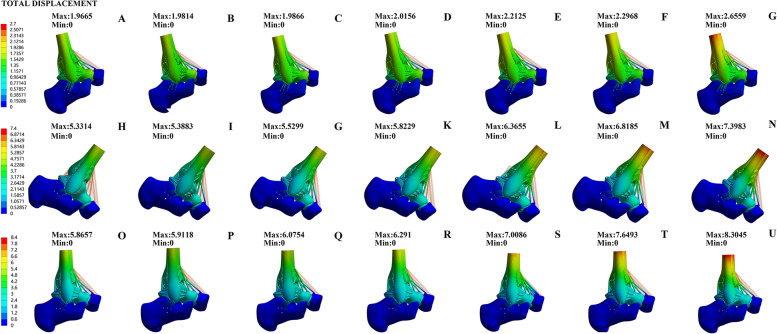
Comparison of displacement of the talus of seven groups in heel-strike phase(A-G),the push-off (H-N) phase, and the midstance phase(O-U): Normal Group (A-H–O); 2 mm* 2 mm defect (B-I-P); 4 mm*4 mm defect (C-J-Q); 6 mm*6 mm defect (D-K-R); 8 mm*8 mm defect (E-L-S); 10 mm*10 mm defect (F-M-T); 12 mm*12 mm defect (G-N-U)

The displacement of the talus (Fig. 6) indicates the overall stability of the ankle joint. Our study found that compared to the normal one, the changes of the talus movement in areas of the OLT defect mainly occurred in the midstance and push-off phases. The talus displacement in the heel-strike phase was small, and the change was not noticeable. The talus displacement was the same as the previous stress indexes in the midstance and push-off phases. When the defect area was less than 6 mm * 6 mm, we found that the difference between the talus displacement and the normal ankle joint was not obvious. The talus displacement of the midstance and push-off phases gradually increased since the defect area at 6 mm * 6 mm. When the defect area was 12 mm * 12 mm, the talus displacements reached maximum values, 8.3045 mm (midstance phase) and 7.3983 mm (push-off phase), which significantly increased compared to those of the normal group, showing a positive growth trend. Therefore, OLT defects have little effect on ankle joint stability when the defect area is less than 6 mm * 6 mm. When the defect area started from 6 mm * 6 mm, it can cause ankle joint instability. We speculate that the reason for this result may be related to the anatomical structure of the talus, as it is wide in the front and narrow in the back. In the heel-strike phase, the ankle joint is in the dorsal position, and the ankle joint is relatively stable, while in the midstance and push-off phases, the ankle joint's stability is reduced the biomechanical impact of the OLT defect is more prominent.

Therefore, for OLT, the impact on joint stress and stability should be actively considered to provide targeted treatment to reduce the damage caused by the defect area to the ankle joint. If the defect area is less than 6 mm* 6 mm, the stress and displacement of the ankle joint are not very obvious. Appropriate treatment should be taken according to the clinical manifestations of the patient, and conservative treatment methods can be considered. Studies have been conducted on the area defect above 6 mm in diameter [33, 34]. However, it has not been reported whether the defect below 6 mm affects the stability of the ankle joint. In our study, stress and displacement increased with increasing OLT defect size. The stress and displacement of the ankle joint increased significantly at 6 mm * 6 mm, suggesting that the stability of the ankle joint was significantly weakened when the defect ≥ 6 mm in diameter and surgical intervention is needed. The stress changes of the ankle joint should be minimized, and the stability of the ankle joint should be restored as soon as possible to reduce the damage to the ankle joint during later weight-bearing activities.

This study has several limitations. First, FEA only simplifies the ankle joint model, and it cannot fully replicate the characteristics of the human ankle joint. Second, the square shape defect may cause concentrated stress, especially at sharp angles. Further biomechanical tests and clinical trials are needed to confirm our findings. Other shapes of the defect will be studied, such as rounding the edges to prevent local stress concentration.We are ready to verify the experimental results of this study in clinical samples or cadaver samples.

## Conclusions

The effect of the size of the OLT defect on the ankle biomechanics is evident, especially in the midstance and push-off phases. When the size of the defect reaches 6 mm * 6 mm, the most noticeable change in the stability of the ankle joint occurs, and the effect does not increase linearly with increasing size of the defect. For the defect area ≥ 6 mm * 6 mm, because the stress and displacement of the ankle joint are showing a positive growth trend, which has a great impact on the stability of the joint, we can consider the surgical treatment.

## Data Availability

The datasets used and analysed during the current study are available from the corresponding author on reasonable request.

## Abbreviations

FEA: Finite element analysis
OLT: Osteochondral lesion of the talus
IGES: Initial Graphics Exchange Specification
HU: Hounsfield Unit
BMS: Bone marrow stimulation
